# Xenon consumption during general surgery: a retrospective observational study

**DOI:** 10.1186/2045-9912-3-12

**Published:** 2013-06-11

**Authors:** Christian Stoppe, Achim Rimek, Rolf Rossaint, Steffen Rex, Ana Stevanovic, Gereon Schälte, Astrid Fahlenkamp, Michael Czaplik, Christian S Bruells, Christian Daviet, Mark Coburn

**Affiliations:** 1 Department of Anaesthesiology, University Hospital, RWTH Aachen, Aachen, Germany; 2 Department of Intensive Care Medicine, University Hospital, RWTH Aachen, Aachen, Germany; 3 Department of Anaesthesiology, University Hospitals Gasthuisberg, KU Leuven, Leuven, Belgium; 4 Air Liquide Santé International, 1 chemin de la Porte des Loges, Les Loges en Josas 78354, France

**Keywords:** Anaesthesia, Xenon, Closed-circuit respirator

## Abstract

**Background:**

High costs still limits the widespread use of xenon in the clinical practice. Therefore, we evaluated xenon consumption of different delivery modes during general surgery.

**Methods:**

A total of 48 patients that underwent general surgery with balanced xenon anaesthesia were retrospectively analysed according to the mode of xenon delivery during maintenance phase (ECO mode, AUTO mode or MANUAL mode).

**Results:**

Xenon consumption was highest during the wash-in phase (9.4 ± 2.1l) and further decreased throughout maintenance of anaesthesia. Comparison of different xenon delivery modes revealed significant reduced xenon consumption during ECO mode (18.5 ± 3.7L (ECO) vs. 24.7 ± 11.5L (AUTO) vs. 29.6 ± 14.3L (MANUAL); p = 0.033). No differences could be detected with regard to anaesthetic depth, oxygenation or performance of anaesthesia.

**Conclusion:**

The closed-circuit respirator Felix Dual offers effective reduction of xenon consumption during general surgery when ECO mode is used.

## Introduction

Official permission for xenon to be used as an anaesthetic was issued by the German Medical Products and Drugs authorities in October 2005 and by the European Medicines Evaluation Agency in March 2007. Since that time a growing body of evidence indicates xenon`s beneficial cardio- and neuroprotective characteristics [1-8]. In addition, xenon offers various characteristics of an ideal anaesthetic gas with a favourable safety profile [9], [10]. In particular its fast recovery after termination of anaesthesia underlines its desirable pharmacodynamics in the clinical practice [11].

Despite these promising findings, the high costs of xenon (about more or less 20€/L) and increased xenon consumption, resulting from high gas concentration needed to achieve target concentration, limit the general use of this auspicious anaesthetic during general anaesthesia [12], [13]. Therefore the challenge of high cost and the favourable properties form an obstacle that necessarily has to be overcome. The combination of recent technologic developments of closed-circuit respirators, the small blood-gas partition coefficient and the low patient uptake in average adults offers promising approaches to reduce gas consumption and the resulting costs [13], [14]. Although the highest xenon consumption is known to occur during the first hour of anaesthesia, effective strategies to counteract this discrepancy are still lacking [15]. The closed-circuit respirator Felix Dual (Felix Dual™, Air Liquide Medical Systems, France) offers encouraging possibilities for an overall effective reduction of gas consumption.

The aim of the present study was to evaluate the potential xenon sparing effects of Felix Dual™ that might be provided by the available xenon delivery mode ECO, AUTO and MANUAL.

Therefore we investigated gas consumption of xenon throughout different periods of anaesthesia and further investigate different modes of delivery. We hypothesize that xenon consumption might be effectively reduced using ECO mode from Felix Dual respirator.

## Methods

### Patients and study design

The present study was part of a large prospective, randomized clinical trial carried out at the University Hospital of the RWTH Aachen between 2008 and 2011. After obtainment of written informed consent, patients enrolled in the trial were initially randomly assigned to one of the two study arms (xenon vs. sevoflurane) and blinded to receiving either sevoflurane or xenon. Data on xenon consumption and administration were recorded automatically in 50 patients. We retrospectively analysed the recorded data from those patients who were consecutively enrolled and randomized to receive xenon anaesthesia.

The study was registered at the EMA (EudraCT number: 2008-004132-20) and at ClinicalTrials.gov (NCT number: 00793663). The local institutional ethics committee and the German federal drug administration (BfArM) approved the study. Ethical approval for this study (EK 110/08) was provided by the Ethical Committee of RWTH Aachen University, Aachen, Germany (Chairperson Prof. G. Schmalzing) on 18. September 2008.

All screened patients were eligible, if they were classified for ASA I-II physical status category and who were undergoing gynaecologic or urologic abdominal surgery. Baseline characteristics (age, sex, height, weight and calculated BMI) were documented separately.

Exclusion criteria were severe cardiac dysfunction (e.g. recent myocardial infarction, heart failure, acute coronary syndrome within the last 24 hours prior to surgery), asthma, acute or chronic obstructive pulmonary dysfunction (COPD), acute or chronic liver or renal failure (creatinine > 1.5 mg/dl), history of hypersensitivity, suspicion of malignant hyperthermia and known or supposed pregnancy.

#### Respirator

Felix Dual is a xenon-capable ventilator. It offers all state-of-art ventilation modes for anaesthesia (volume controlled, pressure controlled and pressure support modes), safe operation at minimal fresh gas flow (0,5 l/min), an integrated sidestream gas monitoring systems for measurements of inspiratory and expiratory gases anaesthetics. Although the circuit was initially controlled at not lower than 0,5 L/min, it is capable to ventilate in closed-circuit and drive intermittently the electronic mixer at very low and precise flow. Furthermore the ventilator allows a flexible inspiratory and expiratory flow measurement and determination of the xenon concentration in the breathing gas.

Felix Dual is designed with two automated xenon administration modes, the ECO and AUTO mode, which are specifically designed to allow conducting of xenon anaesthesia under affordable and safe conditions.

### Induction and maintenance anaesthesia

After admission to the operation theatre standard monitoring of cardiovascular and respiratory function was established (ECG, peripheral Saturation SPO_2_, non-invasive blood pressure (DatexOhmeda AS/3 monitor, GE Healthcare, Helsinki, Finland).

All patients received standardized anaesthesia according to our study protocol. Induction of anaesthesia was performed using propofol (2.0 mg · kg^-1^) and remifentanyl infusion (0.5 μg kg^-1^ · min^-1^) over a period of 60 seconds, followed by a reduction to 0.15 μg · kg^-1^ · min^-1^ if appropriate. Muscle relaxation was obtained with rocuronium bromide (0.6 mg · kg^-1^ bolus for intubation, 0.1 to 0.15 mg · kg^-1^ repetitive boli as needed for anaesthesia and surgery). After tracheal intubation the maintenance of general anaesthesia followed by remifentanil (0.15 μg · kg^-1^ · min^-1^) and (target concentration of 53–62 vol% expiratory xenon). Intraoperative treatment was standardized according to our clinical routine and recorded subsequently (anaesthesia time, opiate, propofol, rocuronium bromideand gas consumption) [16]. Adjustment of anaesthetic depth was performed by adapting end-expiratory xenon concentration depending on significant physiological changes (e.g. difference (>20%) of the baseline arterial pressure or heart rate, increase of inspiratory pressure or expiratory CO_2_ or major change of end-tidal anaesthetic-agent concentration, intermittent spontaneous breathing and/or intolerance of mechanical ventilation, coughing, abdominal pressing, sweating and eye tearing). Additionally bispectral index (BIS VISTA™ monitor, software 2.00, Aspect Medical Systems – Covidien // BIS Model A 2000®, Software Version 2.21, Aspect Medical Systems, Boston, MA, USA) was used for the assessment of adequate anaesthetic depth (recommended reference range for general anaesthesia ranged from 40 to 60.). The monitoring was maintained from arriving in the operation theatre until full recovery after stop of anaesthesia.

At the end of surgery (after spontaneous recovery of neuromuscular block), patients were allowed to breathe 100% oxygen and transferred to the Post Anaesthesia Care Unit after tracheal extubation. Two and 12 hours postoperatively the occurrence of intraoperative awareness was assessed by an independent physician using the Brice questionnaire [17].

### Xenon administration and ventilation modes

The Felix Dual respirator (version mid-2007) allows measuring the xenon concentration by a hot wire sensor in series with the conventional side stream gas monitoring and is compatible with xenon or other inhalation anaesthetics administration.

**Figure 1:**
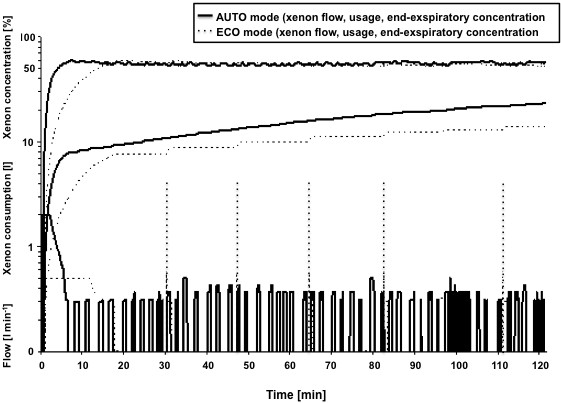
**Schematic illustration for comparison of the automated ECO and AUTO mode.** The figure illustrates the characteristics of the standardized ECO and AUTO mode during wash-in and maintenance, showing xenon injected flow, the resulting expiratory xenon concentration and the overall xenon usage. AUTO mode initially injects xenon at high flow (about 2 L/min) in order to reach fast the target concentration, while the ECO mode initially injects xenon at very low flow (about 0.5 L/min) to be economic. Accordingly during maintenance the AUTO mode repetitively injects xenon with frequent short pulses at low flow while the ECO mode only injects xenon each 10 to 20 minutes during maintenance.

When applying xenon anaesthesia, the first phase was similar to any other general anaesthesia with a classical pre-oxygenation followed by the induction phase with an intravenous agent. After loss of consciousness and intubation of the trachea the denitrogenation started (ventilating with 100% O_2_ with 6 to 12 L/min). The purpose of the denitrogenation was to support exhalation of nitrogen. Denitrogenation was considered as terminated when the expiratory O_2_ concentration reaches 91%-92%. At that time, the Felix Dual could be switched to either automated ECO or AUTO mode. In the ECO mode, during wash-in phase, xenon flow was initially set to 0.5 L/min. While the inspiratory O_2_ concentration progressively decreased the expiratory xenon concentration simultaneously increased until target concentrations were reached. During maintenance phase, xenon injections were automatically performed to maintain regular ventilation with expiratory xenon concentration not lower than 11% below target xenon concentration. In this mode, we expected xenon injections to occur periodically each 10 to 20 minutes depending on O_2_ and Xenon patient uptake or when expiratory concentrations have significantly decreased (Figure 1).

In the AUTO mode, during wash-in phase, the respirator aimed to reach quickly the target concentration. The xenon injection was automatically set at a much higher flow (about 2 L/min) resulting in excess xenon consumption. At the end of wash-in phase, the respirator automatically closed the circuit and started to regulate O_2_ and/or xenon by short-pulsed injections in order to maintain the inspiratory O_2_-concentration to its programmed target. During maintenance phase, Felix Dual alternated short pulsed-injections of O_2_ and xenon in order to compensate O_2_ and xenon patient uptakes but also to continuously eliminate accumulating exhaled strange gases. The AUTO mode aims to maintain stable expiratory xenon concentration in the ± 5% tolerance range (Figure 1).

In addition to the ECO and AUTO mode xenon wash-in and maintenance were also conducted manually by selecting the MANUAL mode. Here, setting of fresh gas flow, O_2_ and xenon concentration were done individually. The MANUAL mode was as well available while conducting the AUTO and ECO mode to wash out accumulated strange gases and compensate significant decreases from target concentration (e.g. in case of long apnoea, long disconnection).

After termination of surgery the xenon application and anaesthesia was finished by activating pure O_2_ flow or O_2_/Air flow. Herein it was even possible to remain either in ECO, AUTO or MANUAL mode respectively. In case of ECO or AUTO mode during recovery, Felix Dual was automatically set at pure O_2_ fresh gas flow (4 L/min). If the recovery phase was managed in MANUAL mode, the time needed for the patient to exhale most part of the accumulated xenon was controlled by setting the appropriate fresh gas flow (about 2–3 minutes with 8 to 12 L/min).

### Choice of Felix Dual Xenon administration modes for the study

Since the Felix Dual respirator was able to deliver xenon in different administration modes, for all of the enrolled patients in the xenon arm, the choice of the xenon administration mode was fully left on the discretion of physician in charge.

For the major data analysis, all patients who received xenon anaesthesia were retrospectively separated into 3 groups according to the delivery mode during maintenance: ECO group, AUTO group and MANUAL group. They were defined as follows:

– ECO group: All patients who received standardized xenon delivery by the means of ECO or AUTO mode during Wash-in and ECO mode during maintenance phase were included in this group.

– AUTO group: All patients who received standardized xenon delivery by the means of AUTO or ECO mode during Wash-in and AUTO mode during Maintenance phase were included in this group.

– MANUAL group: All patients who received not standardized anaesthetic delivery and/or of different delivery modes (i.e. combination of CONTINUOUS, ECO and/or AUTO modes) were subdivided in this group. Therefore this group represents a not-automated and widely inappropriate xenon administration.

### Statistical analysis

All data were statistically analysed using a commercially available software package (SPSS 19.0 (SPSS inc., Chicago, IL, USA).

All enrolled patients that were initially randomized to receive balanced xenon anaesthesia were retrospectively analysed according to the xenon delivery mode during maintenance (ECO, AUTO and MANUAL).

As primary endpoint we investigated the xenon consumption using the different automated AUTO and/or ECO mode of Felix Dual.

Secondary endpoints were the differences between AUTO, ECO and MANUAL groups with respect to xenon flow and gas consumption within wash-in and maintenance. Furthermore we calculated the learning period, which demonstrates the tendency of xenon consumption over a period of 2 years. The linear trend of the learning curve was calculated using a regression analysis and determination of correlation coefficient.

Given the explorative character of our pilot study, the significance level of the fixed-effects results was not adjusted for multiple hypotheses (i.e., for all respiratory variables tested in this investigation).

All data were tested for normal distribution using the Shapiro-Wilk-W-test. Normally distributed results of single measurements were compared using appropriate one-way ANOVA.

Proportions were compared using the Chi-square test. In all cases, a level of p < 0.05 was considered statistically significant.

## Results

### Patients

**Figure 2:**
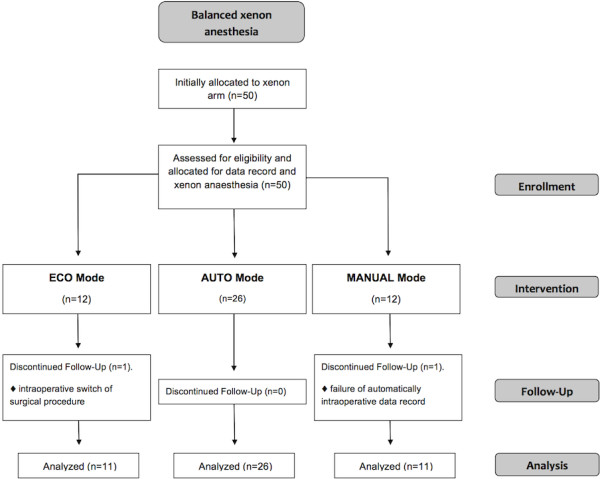
Flowchart of the study.

Data on gas consumption and xenon delivery were recorded in a total of 50 patients. Follow up and final analyses of data were performed in 48 patients. The reasons for the two drops out are due to failure of continuous recording of respiratory data (AUTO group) and intraoperative switch/extend scheduled operative procedure (ECO group) (Figure 2).

Patients treated with ECO, AUTO or MANUAL mode did not differ regarding most relevant baseline characteristics including such as age, gender and body mass index (Table 1). Likewise no difference could be detected with respect to neuraxial anaesthesia, type of surgery, remifentanil, propofol, rocuronium consumption, postoperative analgesia and awareness (Table 2). For the duration of anaesthesia, the BIS values remained within the recommended reference range in all patients.

**Table 1:** Comparison of baseline characteristics and data on surgery

	**All**			**ECO Mode**			**AUTO Mode**			**MANUAL Mode**			***p-value***
	**(n = 48)**			**(n = 11)**			**(n = 26)**			**(n = 11)**			
**Demographic data:**													
Age (years)	46.7	±	12.6	49.0	±	12.3	44.4	±	11.6	49.6	±	15.0	0.405
Sex, male (n, %)	6		(12.5)	2		(18)	3		(12)	1		(9)	0.793
Height (cm)	169.8	±	9.3	174.1	±	8.6	168.1	±	9.6	169.6	±	8.6	0.202
Weight (kg)	75.7	±	17.6	76.2	±	16.3	71.5	±	14.9	84.8	±	22.2	0.109
BMI (kg/m^2^)	26.0	±	5.1	25.0	±	3.4	24.9	±	3.4	29.5	±	8.2	0.129
**ASA classification:**													
ASA I (n)	24		(50)	6		(55)	15		(58)	3		(27)	0.225
ASA II (n)	24		(50)	5		(46)	11		(42)	8		(73)	0.216

Data are presented as mean±SD (normally distributed data) or as absolute numbers (with the percentage (%) of the whole). *BMI*, Body Mass Index.

**Table 2:** Comparison of surgery related data

	**All**			**ECO Mode**			**AUTO Mode**			**MANUAL Mode**			***p-value***
	**(n = 48)**			**(n = 11)**			**(n = 26)**			**(n = 11)**			
**Position:**													
Spine Position (n)	45		(94)	10		(91)	24		(92)	11		(100)	*0.529*
Lithothomy (n)	3		(6)	1		(2)	2		(4)	0		(0)	
**Type of peridural anaesthesia:**													
Lumbal (n)	3		(7)	1		(14)	2		(8)	0		(0)	*0.737*
Thoracal (n)	13		(32)	3		(7)	7		(17)	3		(7)	
**Type of surgery:**													
Gynaecology (n)	31		(65)	6		(55)	19		(63)	6		(55)	*0.159*
Urology (n)	17		(35)	6		(55)	6		(23)	5		(46)	
**Intraoperative data:**													
Anaesthesia time (min)	142	±	44	143	±	32.4	135.0	±	51.0	161.2	±	32.4	*0.258*
Mean remifentanyl consumption (μg/kg/min)	0.16	±	0.07	0.14	±	0.05	0.17	±	0.07	0.17	±	0.08	*0.504*
Propofol consumption (mg)	214.9	±	79.4	180.0	±	71.8	221.3	±	64.3	235.9	±	108	*0.222*
Rocuronium consumption (mg)	39.2	±	9.1	39.0	±	10.9	39.0	±	9.2	40.2	±	7.7	*0.933*
**Postoperative data:**													
Postoperative use of piritramid (mg)	12.2	±	7.7	10.5	±	7.2	14.0	±	7.8	8.2	±	6.6	*0.152*
PACU time (min)	86.8	±	48.5	79.1	±	37.9	91.9	±	57.9	82.0	±	31.1	*0.726*
**Brice questionnaire:**													
2 h post-anaesthesia (n) (awareness/dreams)	0/3		(0/6)	0/1		(0/9)	0/1		(0/4)	0/1		(0/9)	*0.756*
12 h post-anaesthesia (n) (awareness/dreams	0/1		(0/2)	0/0		(0/0)	0/1		(0/4)	0/0		(0/0)	*0.649*

Data are presented as mean±SD (normally distributed data) or as absolute numbers (with the percentage (%) of the whole group). *PACU*, post-anaesthesia care unit.

### Illustration of Full-ECO and Full-AUTO modes

**Figure 3:**
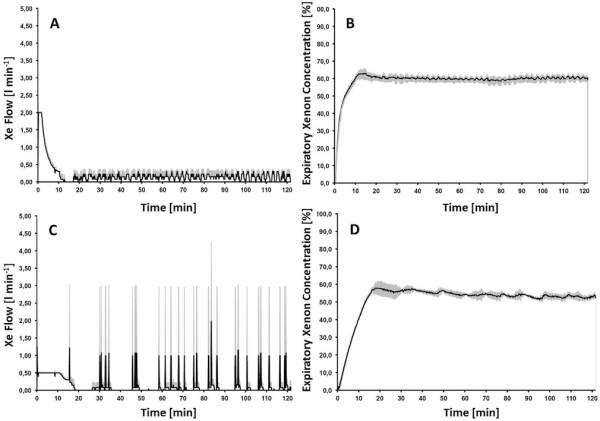
**Comparison of ideal groups.** Full-ECO (n = 4) and Full-AUTO (n = 4) delivery mode by the means of xenon flow and end-expiratory xenon concentration throughout xenon application. Data are presented as mean ± standard deviation. **A** and **B** corresponds to Full-AUTO mode. **C** and **D** corresponds to Full-ECO mode.

Since the choice of delivery mode was left to the discretion of the performing anaesthesiologist, the applied delivery mode was not standardized. Patients were retrospectively separated into 3 groups according to their xenon delivery mode during maintenance. To identify the real difference between ECO and AUTO mode we compared two small patient groups that received either one of both delivery modes throughout wash-in and maintenance. The total xenon consumption was significantly reduced in the Full-ECO group in contrast to Full-AUTO group (Table 3). Accordingly, the xenon consumption per hour and mean end expiratory xenon concentration were significantly reduced in the Full-ECO group (Figure 3A-D).

**Table 3:** Comparison of ideal group that obtained same mode during wash-in and maintenance

	**Full-ECO mode**			**Full-AUTO mode**			**p-value**
	**(n = 4)**			**(n = 4)**			
**Xenon wash-in phase:**							
Duration of Wash-in (min)	16.8	±	1.0^§§^	11.3	±	1.3	***0.000***
Total xenon consumption (l)	7.8	±	0.5^§§^	9.6	±	0.9	***0.010***
**Maintenance of anaesthesia:**							
Time of maintenance (min)	128.5	±	10.7	138.3	±	9.2	0.217
Total xenon consumption (l)	12.0	±	3.6^§^	18.2	±	3.1	***0.041***
Mean consumption (l/hour)	5.5	±	1.4^§^	7.8	±	0.9	***0.034***
Mean FiO_2_ concentration (Vol%)	34.8	±	0.8	33.1	±	1.5	0.092
Mean Expiratory Xe concentr. (Vol%)	54.3	±	1.4^§§^	60	±	1.3	***0.001***
**Overall xenon consumption:**							
Xenon usage 1st hour (l/min)	0.181	±	0.021^§^	0.256	±	0.025	**0.018**
Xenon usage 2nd hour (l/min)	0.115	±	0.034	0.141	±	0.012	0.198
Xenon usage 3rd hour (l/min)	0.078	±	0.018	0.128	±	0.025	0.290
Time of xenon application (min)	145	±	10.5	149.5	±	8.3	0.217
Mean xenon consumption (l/hour)	8.2	±	1.3^§§^	11.1	±	0.5	***0.041***
Total xenon consumption (l)	19.8	±	3.8^§§^	27.8	±	2.4	***0.037***

Data are presented as as mean±SD (normally distributed data). Bold italic P-values indicate significant difference between the groups (p < 0.05), §(§) = p < 0.05(0.01) vs. Full-AUTO mode.

In contrast to this idealized group classification (n = 8), remaining patients (n = 40) showed different delivery-modes during wash-in and maintenance. Therefore we separated patients into 3 different groups according to the applied delivery mode during maintenance.

### Denitrogenation and wash-in of xenon

The mean duration of pre-oxygenation and denitrogenation (time between first expiratory CO_2_ detection and start of xenon) in all patients is 9.3 ± 4.3 minutes with no significant differences between the 3 groups.

The duration of wash-in was prolonged in the ECO group (13.7 ± 2.8 min) and differed significantly from the other groups (p = 0.002). In contrast the total xenon consumption during wash-in was comparable between ECO and AUTO group and significantly lower than in the MANUAL group (Table 3). Considering the mean xenon consumption throughout wash-in in all patients, the xenon usage was high when compared to the mean overall usage (39%), while it only represented 9% of the mean total duration of xenon application (Table 4).

**Table 4:** Intraoperative data on xenon consumption during wash-in, maintenance and overall consumption

	**All**			**ECO Mode**			**AUTO Mode**			**MANUAL Mode**			***p-value***
	**(n = 48)**			**(n = 11)**			**(n = 26 )**			**(n = 11 )**			
**Xenon wash-in phase:**													
Duration of wash-in (min)	11.5	±	2.7	13.7	±	2.8	11.0	±	1.7^§^	10.2	±	3.2^§§^	***0.002***
Denitrogenation (min)	9.3	±	4.3	8.1	±	4.7	9.6	±	3.4	9.9	±	5.8	*0.556*
Total xenon consumption (l)	9.4	±	2.1	8.6	±	0.9	8.9	±	0.9	11.1	±	3.7*^§§^	***0.005***
**Maintenance of anaesthesia:**													
Time of maintenance (min)	115	±	44	111	±	30	113	±	53	123	±	30	*0.781*
Total xenon consumption (l)	15.0	±	11.1	9.9	±	3.6	15.7	±	11.9	18.5	±	12.8	*0.172*
Mean xenon usage (l/hour)	7.5	±	3.6	5.2	±	1.1	7.8	±	2.6^§^	8.9	±	6.0^§^	***0.049***
Mean FiO_2_ concentration (Vol%)	34.6	±	2.6	34.9	±	1.4	33.8	±	2.6	36.2	±	2.7*	***0.029***
Mean expiratory xenon concentration	58.2	±	3.0	55.9	±	2.4	59.7	±	2.7^§§^	57.0	±	2.5*	***0.000***
**Overall xenon consumption:**													
Time of **anaesthesia** (min)	142.8	±	44.1	142.6	±	32.4	135.0	±	51.0	161.3	±	32.4	*0.258*
Time of xenon application (min)	126	±	44	125	±	32	124	±	53	133	±	30	*0.842*
Xenon usage 1st hour (l/min)	0.268	±	0.121	0.205	±	0.030	0.263	±	0.082	0.347	±	0.202 ^§^	***0.020***
Xenon usage 2nd hour (l/min)	0.128	±	0.042	0.098	±	0.030	0.143	±	0.042^§^	0.115	±	0.035	***0.007***
Xenon usage 3rd hour (l/min)	0.098	±	0.056	0.080	±	0.043	0.112	±	0.050^§^	0.085	±	0.077	***0.042***
Mean xenon consumption (l/hour)	11.9	±	4.0	9.2	±	1.9	12.5	±	2.6	13.4	±	6.7^§^	***0.031***
Total xenon consumption (l)	24.4	±	11.4	18.5	±	3.7	24.7	±	11.5	29.6	±	14.3^§^	***0.033***

Data are presented as mean ± SD (normally distributed data). Bold italic P-values indicate significant difference between the groups (p > 0.05), §(§) = p < 0.05(0.01) vs. ECO, *(*) = p < 0.05(0.01) vs. AUTO.

The high xenon consumption becomes obvious when comparing the xenon usage in all patients during the 1st hour (that includes induction of anaesthesia) with the following 2nd and 3rd hour of xenon application (1st min: 0.268 ± 0.121L/hour vs. 2nd min: 0.128 ± 0.042L/hour; p = 0.000 and vs. 3rd hour 0.098 ± 0.056L/min; p = 0.000).

### Maintenance of xenon anaesthesia

The overall duration of xenon application, xenon usage and the corresponding xenon usage per hour are depicted in Table 3. After completion of 1st hour, xenon usage continuously decreased during maintenance of anaesthesia in all patients. While application time of xenon was comparable between the groups, total xenon consumption during maintenance showed a tendency of being lowest in the ECO group (p = 0.172), becoming significant when comparing the hourly xenon usage (p = 0.049). Comparison of hourly xenon usage during 1st, 2nd and 3rd hour between the groups revealed a lowest xenon usage in the ECO group that significantly differed from MANUAL group (1st hour) and from AUTO group (2nd and 3rd hour of maintenance) (Table 4). Accordingly the overall xenon consumption was lowest in the ECO group throughout xenon application and showed a significant difference to the MANUAL group. Likewise the mean expiratory xenon concentration in the ECO group was significantly reduced in comparison to AUTO and MANUAL group (55.9 ± 2.4vol% (ECO) vs. 59.7 ± 2.7vol% (AUTO) vs. 57.0 ± 2.5vol% (MANUAL)); p = 0.000). Interestingly data for either mean xenon concentration, xenon usage per hour or mean xenon consumption showed reduced standard deviation compared to the AUTO or MANUAL group reflecting the well-standardized procedure of ECO mode (Table 4).

### Learning curve

**Figure 4:**
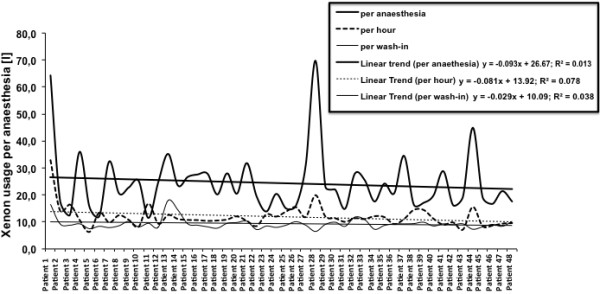
Illustration of the learning process within the entire observation period (24 month) in all patients.

Figure 4 illustrates the learning curve that has been recorded between February 2009 to November 2010 in 48 patients. The linear graphic represents the evolution of xenon consumption and shows a visual tendency towards reduction of gas consumption. Within two years we observed a decrease in xenon consumption of −4.6 L in overall quantity per anaesthesia and of −3.9 L per hour.

Present learning curve has been constantly developed by self-implication and self-dedication of the anaesthesiology stuff with no particular training or specific refreshing courses since the initial training provided in 2008.

### Overall xenon consumption

The total xenon consumption in 48 patients was 1170 L that nearly equals to the volume of one LENOXe B10 cylinder. The overall xenon consumption corresponds to about 101 hours of anaesthesia (mean duration: 2 hours) that can be subdivided into 54 hours in AUTO Mode, 24 hours MANUAL Mode and 23 hours in ECO Mode.

## Discussion

The results of the present study showed that for the same depth and duration of anaesthesia xenon consumption can effectively be reduced by using the xenon delivery mode ECO of Felix Dual respirator during general surgery.

Today an increasing body of evidence indicates the numerous favourable effects since the introduction of xenon in clinical practice in 1990 [18]. While high costs and restricted availability still limit the introduction and general use of xenon into clinical practice, various promising technical advances were investigated for an effective reduction of xenon waste. [15], [19], [20]. In view of low patient uptake of xenon (about 2.5-4l/h), closed-circuit respirators have been developed and adapted in the past decade in order to provide an economical xenon delivery [21].

Since 2007–2008 (depending on countries), the commercially available closed-circuit respirator Felix Dual belongs to the few systems that are capable to provide xenon anaesthesia in the clinical practice. Today Felix Dual is distinguished by a limited but real clinical use in the daily practice as well as in completed or currently running large multicentre studies investigating the effect of xenon on outcome of patients [clinicaltrails.gov identifier: NCT00919126 (HERACLITE), NCT01199276 (HIPELD), NCT01120405 (CARVASAXe), NCT01294163 (ON-PUMP CABG), NCT01167803 (OBESE PHRC), NCT01262729 (Xenon-MTH-study), NCT01285271 (CARDIAX)]. Although increasing experimental and clinical evidence promotes the use of balanced xenon anaesthesia in various clinical settings, data about technical progress or xenon sparing effects of xenon respirators are still sparse.

Felix Dual offers encouraging technical innovations - more precisely an automated AUTO and ECO xenon delivery mode aiming to reduce an excessive waste of xenon. Therefore we investigated and compared the present delivery modes with the not-automated MANUAL mode in order to reveal any potentially sparing strategies. Since use of the 3 delivery modes were not standardized and left to the discretion of performing anaesthesiologist, we classified the enrolled patients according to their delivery mode during maintenance for final analysis. However, we identified two groups of patients that received either full ECO or AUTO mode for wash-in and maintenance and compared the major characteristics. Herein the comparison of xenon during wash-in and maintenance as well as the xenon usage per hour indicated a significantly reduced xenon consumption in the ECO application mode. In addition the mean end-expiratory xenon concentration was significantly decreased in the Full-ECO group and together with xenon flow did not show major changes (low standard deviation) during the duration, indicating the robust delivery process.

Previous studies repeatedly demonstrated that consumption and/or waste of xenon was remarkably elevated within the wash-in period aiming to remove accumulated nitrogen and accelerate time to target concentration of xenon [15], [22]. It`s important to note that a significant and irreducible part of xenon used during wash-in corresponds to the xenon volume needed to saturate the fast compartment of the patient`s body (several litres of xenon) as well as to saturate the respirator internal volume and tubes (estimated to 3l xenon). While xenon anaesthesia needs to target approximately 60% expiratory concentration with inspiratory O_2_ content not lower than 30%, adequate denitrogenation of patients is basically important to eliminate as much as possible nitrogen that is trapped in the body. However, time of pre-oxygenation (before intubation) and denitrogenation (after intubation) was comparable between the groups. While use of high-flow is frequently necessary during wash-in period, previous studies already supposed induction period to offer most potential to reduce waste of xenon [15], [22].

Recent findings are in line with our findings and total xenon consumption of all patients was apparently highest during wash-in and 1st hour of xenon anaesthesia, when compared to maintenance. Xenon usage significantly decreased throughout further application. Considering the xenon usage within the separated application groups showed that xenon consumption within the 1st hour was lowest in the ECO group. This mode was specially designed to inject xenon at an appropriate low flow that corresponds to xenon and O_2_ patient uptake. Furthermore the ECO mode provided the lowest xenon application during maintenance of anaesthesia resulting in a most reduced total xenon consumption, which is independent from duration of application. Accordingly, the patients in the ECO group exhibited lowest overall xenon consumption throughout the entire xenon application period, which significantly differed to the not-standardized and not-automated MANUAL mode.

Interestingly we observed a significantly reduced expiratory xenon concentration in ECO mode (in comparison to AUTO and MANUAL mode) that might further indicate a xenon sparing effect. Despite these promising sparing effects of ECO mode, the reduced xenon flow might pose a serious challenge when fast adaption of expiratory xenon concentration is needed to maintain an adequate anaesthetic depth. However, the recorded BIS values in al patients were within the recommended reference range and did not reveal any signs of major variation. Likewise postoperative evaluation of intraoperative awareness by the means of Brice questionnaire were negative [17].

In view of a significant reduced usage of xenon in the ECO mode, which was most pronounced in the full ECO group, this delivery mode may provide a promising strategy for an effective costs reduction per anaesthesia. Assuming a price of more or less 20 € for 1L xenon (frequently announced on congresses for LENOXe anaesthesia licensed xenon, Air Liquide, France) we calculated a cost reduction from 592 € to 370 € when compared to the not-automated MANUAL mode. Given the presented learning curve, showing a constant decrease of the overall and hourly xenon consumption, these findings might as well indicate a promising approach for effective cost reduction. Since this development based alone on the experience of involved anaesthesiologists, it remains speculative, if an additional training or periodic refreshing courses would have further improved the performance of the anaesthesiologists team.

Of note, while conducting this observational study, further improvements have been integrated in Felix Dual and a new software upgrade is available since 2011, which aims to further reduce xenon consumption in ECO mode and to improve the precision of AUTO mode. Therefore present results could even be reinforced by the new software version of Felix Dual. However, further studies would be needed to evaluate current progress in xenon reduction.

We have to acknowledge that previous studies already demonstrated less xenon consumption throughout induction and maintenance of anaesthesia [15]. The reasons for that might be due to the single use of xenon as hypnotic agent without additional use of propofol. Likewise the target concentration of previous studies was less than the chosen concentration in the present study.

The present results have to be viewed in the light of a limited retrospective, observational study. Therefore we acknowledge that allocation of patients into ECO, AUTO and MANUAL groups was left to the discretion of performing physician and was not randomized. Furthermore sample size of patients might not be adequately powered and therefore present results have to be interpreted cautiously and viewed as only hypothesis generating. However, the present findings indicate a promising approach for an effective reduction of xenon waste during general anaesthesia. Further studies in larger patient population are needed to investigate this promising approach and encourage further technical developments that might render xenon`s further introduction into clinical use.

In conclusion, the closed-circuit respirator Felix dual offers a promising approach to effectively control xenon consumption in patients undergoing general surgery. The delivery mode ECO showed lowest usage of xenon that was independent from duration of xenon application or applied mean xenon dosage. In comparison to not-automated application by the means of MANUAL mode, the use of ECO mode resulted in about 40% reduction of xenon consumption throughout entire xenon anaesthesia.

## Competing interests

MC and RR received lecture and consultant fees from Air Liquide Santé International, a company interested in developing clinical applications for medical gases, including xenon. For the remaining authors no competing interests exist.

## Authors’ contributions

CS, AR and MCo were equally involved in the development, performance, analysis and together with RR and SR wrote the manuscript. AF, GS, AS, MCz, CB carried out the data acquisition and analysed the preliminary data. The corresponding author takes responsibility for the integrity and the accuracy of the data analysis and had final responsibility for the decision to submit for publication. CD, as an engineer who, in his former position in Air Liquide Medical Systems, developed with his team the Felix Dual ventilator, was mainly involved in processing raw data that have been recovered from Felix Dual as well as in providing needed technical information on Felix Dual concept and use principles. All authors read and approved the final manuscript.
